# Is the Efficacy and Safety of Retrograde Flexible Ureteroscopy in the Elderly Population Different from Non-elderly Adults?

**DOI:** 10.7759/cureus.4852

**Published:** 2019-06-06

**Authors:** Mehmet Caglar Cakici, Sercan Sari, Volkan Selmi, Fatih Sandikci, Nihat Karakoyunlu, Ugur Ozok

**Affiliations:** 1 Urology, Medeniyet University, Göztepe Training and Research Hospital, Istanbul, TUR; 2 Urology, Bozok University Faculty of Medicine, Yozgat, TUR; 3 Urology, University of Health Sciences Diskapi Yildirim Beyazit Training and Research Hospital, Ankara, TUR; 4 Urology, Karabuk University School of Medicine, Karabük, TUR

**Keywords:** ureteroscopy, elderly, efficacy, flexible, kidney stone, ureterorenoscopy, rirs

## Abstract

Background and objectives

The population of elderly adults is increasing globally, and due to metabolic changes related to advanced age, many elderly adults experience kidney stones. Flexible ureteroscopy (f-URS) is a minimally invasive procedure to treat kidney stones, but it is not free of complications. The goals of this study were to analyze the efficacy and safety of f-URS in the management of kidney stones in patients aged ≥60 years and compare the outcomes of this surgery with the outcomes of the same surgery in a younger population.

Materials and methods

We retrospectively reviewed patient data from 1750 patients who met our inclusion criteria and received f-URS at the urology clinic of our hospital from 2012 to 2017. Patients were assigned into two groups: those aged ≥60 years (Group 1, n=291) and those aged 19-59 years (Group 2, n=1459). The perioperative results were evaluated comparatively. We performed multivariable analyses for factors predicting complications.

Results

When we compared the groups on demographic attributes, we noted statistically significant differences in gender, body mass index (BMI), and American Society of Anesthesiologists scores. Stone size and operation time were higher in the ≥60-year age group (Group 1). Other stone characteristics and operative features were similar. Stone-free rates (SFR) after the first procedure were 88.0% in Group 1 and 89.2% in Group 2. SFR and success rates at three months were similar for both groups. The complication rates were similar, and multivariable regression analysis revealed the most important factor affecting the complications was the presence of residual stones in both groups. The second most important factor affecting the complication was the operation time in Group 1 and the number of stones in Group 2.

Conclusion

In our study, there were no significant differences in terms of results and complications among elderly and young patients after f-URS except for the duration of the operation. The prolongation of operation time results in worse outcomes in terms of perioperative complications in patients aged ≥60 years. f-URS is a relatively safe and efficient procedure, with a small risk of minor complications even in the elderly population, with increased comorbidity.

## Introduction

The elderly population (i.e., the group of patients aged 60 years or older) is growing all over the world. Demographic estimates show that in 2050, the human population aged 60 years or older will exceed 2 billion, up from 900 million in 2015. Between 2015 and 2050, the proportion of the world's population aged over 60 years will nearly double from 12% to 22% [1-2]. Stone disease is a significant problem due to the metabolic changes associated with advanced age [3]. Surgery choices to treat stone disease are especially important in elderly patients because of the increase in comorbidity and age-related impairment associated with this population. Although flexible ureterorenoscopy (f-URS) is a minimally invasive procedure for kidney stone treatment, it is not free of complications. The goals of this study were to analyze the efficacy and safety of f-URS in the management of kidney stones in patients aged ≥60 years and compare the outcomes of this surgery with the outcomes of f-URS in a younger population.

## Materials and methods

We conducted a retrospective review of patient data from patients who underwent f-URS at the urology clinic of our hospital from 2012 to 2017. Patients who had sterile preoperative urine cultures were included in the study. Patients with confirmed stone-free rates (SFR) via non-contrast computed tomography (CT) or ultrasound at the first and third postoperative months were included in the study. We excluded patients who were younger than age 18, and those with kidney abnormalities, infection before operation or bleeding disorders. Of the 1,889 patient records assessed, 1,750 patients met the inclusion criteria and were included in the study. Patients were assigned into two groups. Group 1 consisted of patients older than 59 years (n=291), and Group 2 consisted of patients aged 19 to 59 years (n=1,459). Stone sizes were calculated as the longest diameter on non-contrast CT. We compared the groups according to the characteristics of patient demographics, American Society of Anesthesiologists (ASA) score, intraoperative parameters, complications, and postoperative data. Patients who had residues of <2 mm or manifested stone-free status three months postoperatively were recorded as successful outcomes. These patients were considered stone-free.

Statistical analysis

We used IBM SPSS Statistics for Windows, Version 22.0 (IBM Corp, Armonk, NY, US) to analyze the data. The one-sample Kolmogorov-Smirnov test was applied to the variables with quantitative values. We applied the t-test for the variables of age, BMI, and stone burden that had a normal distribution, and the Mann-Whitney test was utilized for other factors. The chi-square and Fisher’s exact tests were used to evaluate the association between the categorical variables. Univariable and multivariable analyses were performed for factors predicting complications. The statistical significance was defined as p<0.05.

## Results

A total of 291 patients (159 men, 132 women) were placed in Group 1, which had a mean patient age of 67±6 years and mean BMI of 26.73±3.5 kg/m2. A total of 1,459 patients (976 men, 483 women; p<0.001) were placed in Group 2, which had a mean patient age of 42±10 years and mean BMI of 25.67±3.3 kg/m2 (p<0.001). More patients in Group 1 had an ASA score of three than Group 2. Stone laterality and localizations were similar in both groups, and a similar amount of lower calyceal stones were observed in both groups. Group 1 had 1.43±0.6 stones with a mean stone size of 16.23±7.5 mm. Group 2 had 1.43±0.7 stones (p=0.268) with a mean stone size of 14.51±7.0 mm (p<0.001; Table 1).

**Table 1:** Demographic and stone characteristics SD: Standard Deviation; BMI: Body Mass Index; ASA: American Society of Anesthesiologists; UPJ: Ureteropelvic Junction

	Group 1 (n=291)	Group 2 (n=1459)	P-value
Age (mean ± SD) (range in years)	67.20 ± 6.1	42.09 ± 10.2	< 0.001
Age (range in years)	60-86	19-59	
Gender, n (%)			< 0.001
Male	159 (54.6)	976 (66.9)	
Female	132 (45.4)	483 (33.1)	
BMI (kg/m^2^)	26.73 ± 3.5	25.67 ± 3.3	< 0.001
ASA score, n (%)			< 0.001
ASA 1	18 (6.2)	554 (38.0)	
ASA 2	176 (60.5)	867 (59.4)	
ASA 3	97 (33.3)	38 (2.6)	
Stone laterality, n (%)			0.325
Right	137 (47.1)	733 (50.2)	
Left	154 (52.9)	726 (49.8)	
Stone localization, n (%)			0.388
Upper calyx	16 (5.5)	85 (5.8)	
Middle calyx	34 (11.7)	149 (10.2)	
Lower calyx	81 (27.8)	362 (24.8)	
Renal pelvis	56 (19.2)	288 (19.7)	
UPJ	50 (17.2)	327 (22.4)	
Multiple	54 (18.6)	248 (17.0)	
Number of stones (mean ± SD)	1.43 ± 0.6	1.43 ± 0.7	0.268
Stone size, mm (mean ± SD)	16.23 ± 7.5	14.51 ± 7.0	< 0.001

The preoperative double-J stent (DJS) usage ratio was higher in Group 1 (p=0.128). The mean operation time was 49.01±18.1 minutes in Group 1 and 45.33±16.9 minutes in Group 2 (p<0.001). Fluoroscopy duration was 33.65±42.8 seconds in Group 1 and 34.33±34.7 seconds in Group 2 (p=0.614). We found no difference in the duration of hospitalization between Groups 1 and 2.

SFR after the first procedure was achieved in 256 patients (88.0%) in Group 1 and in 1,301 patients (89.2%) in Group 2 (p=0.551). Of the 35 patients with residual stones in Group 1, 18 were treated with a second session of f-URS, two were treated with percutaneous nephrolithotomy (PNL), four with shock wave lithotripsy (SWL), and 11 patients were monitored via follow-up with no procedure. In Group 2, among 158 patients with residual stones, 75 patients were treated with a second session of f-URS, three were treated with PNL, 16 were treated with SWL, and 64 patients were monitored via follow-up with no procedure. When the patients were re-evaluated for stone-free status, the success rate was 92.1% in Group 1 and 92.6% in Group 2 at the third-month follow-up (p=0.767; Table 2).

**Table 2:** Perioperative parameters DJS: Double-J Stent; SD: Standard Deviation; SFR: Stone-free Rate

	Group 1 (n=291)	Group 2 (n=1459)	P-value
Preoperative DJS, n (%)			0.128
Prestented	34 (11.7)	129 (8.66)	
Not prestented	257 (88.3)	1361 (90.5)	
Operation time, min (mean ± SD)	49.01 ± 18.1	45.33 ± 16.9	<0.001
Fluoroscopy time, sec (mean ± SD)	33.65 ± 42.8	34.33 ± 34.7	0.614
Ureteral access sheat usage, n (%)	247 (84.9)	1195 (81.9)	0.224
Postoperative DJS usage	255 (87.6)	1212 (83.1)	0.054
Duration of hospitalization, day (mean ± SD)	1.24 ± 1.5	1.16 ± 0.8	0.700
Duration of hospitalization, day (range)	1-15	1-14	
SFR, n (%)	256 (88.0)	1301 (89.2)	0.551
Success rate, n (%)	268 (92.1)	1351 (92.6)	0.767
Complications, n (%)	34 (11.7)	165 (11.3)	0.854

Thirty-four patients (11.7%) had complications related to the operation in Group 1, and one patient died. In Group 2, 165 patients (11.3%) had complications (p=0.854). The more common complications were fever, bleeding, and urinary tract infections in both groups (Table 3).

**Table 3:** Complications according to Satava and Clavien classification systems

	Group 1 (n=291)	Group 2 (n=1459)	Satava Grade	Clavien Grade
Complications, n (%)	34 (11.7)	165 (11.3)		
Fever	10 (3.4)	72 (4.9)		I
Bleeding	6 (2.1)	28 (1.9)	I	I,II
intraoperative	2 (0.7)	11 (0.8)	I	
postoperative	4 (1.4)	20 (1.4)		I
Bleeding-Need transfusion	2 (0.7)	5 (0.3)		II
Urinary tract infection	7 (2.4)	34 (2.3)		II
Perforation	1 (0.3)	6 (0.4)	IIa-b	I,IIIa
Perirenal abscess	1 (0.3)	1 (0.07)		IIIa
Stent migration	2 (0.7)	11 (0.8)		IIIb
Steinstrasse	3 (1.0)	7 (0.5)		IIIb
Sepsis	1 (0.3)	1 (0.07)		IVb
Death	1 (0.3)	0		V

In a multivariate logistic regression analysis, operation time and residual stone presence were significant predictive factors for complications in Group 1 (p<0.001; Table 4).

**Table 4:** Multivariate logistic regression analysis of demonstrating factors predicting complications for Group 1 (n=291) OR: Odds Ratio; CI: Confidence Interval; BMI: Body Mass Index; ASA: American Society of Anesthesiologists; DJS: Double-J Stent

Variables	Univariate Model						Multivariate Model				
	OR	95% CI			P value		OR	95% CI			P value
Age	1,03	0,98	-	1,09	0.284						
Gender	1,61	0,79	-	3,32	0.193						
BMI	1,01	0,91	-	1,12	0.900						
ASA	0,98	0,52	-	1,84	0.941						
Stone localization	1,07	0,84	-	1,37	0.570						
Number of stone	1,05	0,59	-	1,87	0.878						
Stone size	1,03	0,99	-	1,08	0.145						
Usage of access sheat	0,96	0.35	-	2.64	0.943						
Operation time	1.03	1.01	-	1.05	0.003		1.03	1.01	-	1.04	0.004
Fluoroscopy time	0.99	0.98	-	1.01	0.762						
Postoperative DJS	1.26	0.45	-	3.49	0.661						
Residual stone	2.62	1.08	-	6.36	0.033		2.55	1.03	-	6.30	0.043

In Group 2, the multivariable logistic regression analysis showed that the number of stones and residual stone presence were the significant predictors of complications (p<0.001; Table 5).

**Table 5:** Multivariate logistic regression analysis of demonstrating factors predicting complications for Group 2 (n=1459) OR: Odds Ratio; CI: Confidence Interval; BMI: Body Mass Index; ASA: American Society of Anesthesiologists; DJS: Double-J Stent

Variables	Univariate Model						Multivariate Model				
	OR	95% CI			P value		OR	95% CI			P value
Age	1,01	0,99	-	1,03	0.309						
Gender	0.89	0.63	-	1.27	0.525						
BMI	0.97	0.92	-	1.02	0.285						
ASA	0.77	0.57	-	1.05	0.097						
Stone localization	1.07	0.95	-	1.20	0.266						
Number of stone	1.57	1.30	-	1.89	<0.001		1.44	1.19	-	1.75	<0.001
Stone size	1.06	1.04	-	1.08	<0.001						
Usage of access sheat	0,71	0.45	-	1.12	0.143						
Operation time	1.02	1.01	-	1.03	<0.001						
Fluoroscopy time	1.004	1.000	-	1.008	0.036						
Postoperative DJS	0.61	0.37	-	1.01	0.051						
Residual stone	3.62	2.43	-	5.38	<0.001		3.17	2.11	-	4.77	<0.001

## Discussion

Although aging is not a disease, it is a critical factor that affects the functions of organs and the complications and outcomes of the operation. Urolithiasis is a significant problem due to the formation of some metabolic and lifestyle changes in the elderly population. As in all age groups, especially in developing countries, the incidence of stone disease is increasing in the elderly population. Some studies showed that about 10% to 15% of patients who have stone disease are members of the elderly population [3-4]. Risk factors for kidney stones include metabolic parameters, dietary factors, and chronic conditions such as hypertension and obesity [5-7]. Obesity is one of the independent risk factors for urolithiasis, and its incidence increases with aging [8]. In our study, BMI was significantly higher in the elderly population. Factors such as increased sedentary lifestyle and the impact of chronic diseases are the first accused factors in the development of this condition. Sumner et al. reported that the frequency of obesity and metabolic syndrome increased due to aging [9]. The male-to-female ratio was 2:1 in the young group who underwent f-URS operation; this ratio is almost equal in the elderly population. Unlike the young group, the sedentary lifestyle of older women in our society is relatively higher so female patients in the elderly group may be on the rise. Due to the increase in chronic comorbidities in the elderly population, the number of patients with the ASA-3 score is more common, statistically.

Higher BMI, relative sedentary lifestyle, and metabolic factors may have led to the greater detection of stone sizes of patients in Group 1. In a study performed by Berardinelli et al. with patients who underwent f-URS, they compared those older than 65 years with those younger than 65 years and found that the operation time was similar between both groups [10]. Stone sizes were similar in this study. In contrast, in our study, the stone size was bigger in the elderly population, and the operative time was longer. However, the duration of fluoroscopy was similar in both groups. We used a ureteral access sheath to decrease the intrarenal pressure and to reduce the surgical burden for most patients. Both groups were not statistically different. In the current study, postoperative DJS was used in 87.6% and 83.1% of cases, and this result was similar to reports in the current literature [11-12]. In the older group, stone sizes and operation times were statistically greater than in the younger group. Other perioperative outcomes were not influenced by age.

f-URS has an almost higher SFR than SWL and lower morbidity than PNL [13]. In our study, the overall SFR after a single procedure was 88.0% and 89.2% for Groups 1 and 2, respectively. Akman et al. reported that initial SFR was 82.1% in their study in elderly patients [14]. In a prospective study in which patients older than 70 years were monitored via follow-up for five years, the SFR was 88.0% [15]. In the general population, the SFR of f-URS in the literature varies between 65% and 95% [16-18]. As a result of the large number of cases and more experience in our clinic, our success rate was higher than in most centers. The success rate of patients re-evaluated after additional procedures or watchful waiting at the three-month follow-up were 92.1% and 92.6% in Group 1 and Group 2, respectively. Considering these results, f-URS is a treatment alternative that can be applied with high success rates in the elderly population with kidney stones.

Clavien and Dindo recommended a system for grading the severity of postoperative complications [19]. This classification is a simplistic and applicable grading system of postoperative complications. According to this classification, the majority of complications in both groups were grade I-II, indicating that this procedure had minimal impact on the patient. Even in the elderly, the fact that grade III-IV complications are below 3% reinforces this situation. Most complications in elderly patients with high comorbidity and anesthesia risk, as in young people, are based on the Grade I-II of classification of modified the Clavien and/or Grade I-II of the Satava system [19-21]. The multivariate logistic regression analysis of demonstrating factors predicting complications showed that the presence of residual stone and operation time are the most crucial factor in the elderly group. When the operation time is prolonged, perioperative complications increase. In contrast, operation time did not correlate with the occurrence of complications on multivariable analyses in Group 2. In this young group, factors affecting the complications were determined as the presence of residual stone and number of stones. These factors in the population under the age of 60 may be effective factors in the general population. Increased age and comorbidities in the elderly population do not increase the complication rate associated with f-URS surgery. Berardinelli et al. reported that the complication rates were similar between the elderly and young patients who underwent f-URS (9.89% vs. 14.28%, respectively) [10]. In a study conducted in elderly patients, the rate of complications associated with f-URS in the 65- to 74-year age group and patients aged 75 years or older were reported as 11.9% vs. 12.8%, respectively [22]. The complication rates in our study were similar to those found in the literature.

Our study has some limitations. First, this was a retrospective study. We determined the age threshold between groups was 60 years to align with the same threshold seen widely in the literature. The definition of elderly people is largely variable due to varying retirement ages and life expectancies seen in different countries. Therefore, the data for the elderly are heterogeneous, and the results are difficult to compare. A match-pair analysis on the subject will contribute to the literature. Also, the lack of a standardized SFR definition makes it difficult to compare our findings across many studies. f-URS studies should focus on standardized results and should be performed in a multi-institutional evaluation.

## Conclusions

Stone disease and treatment in the elderly population is a real and growing concern, given the growing elderly population. In our study, there were no significant differences in terms of results and complications among elderly and young patients after f-URS except for the duration of the operation. In this group with high comorbidity, this f-URS is an effective and safe treatment method without the excessive length of operation. The prolongation of operation time results in worse outcomes in terms of perioperative complications in the elderly population as compared to younger patients. f-URS is a safe and effective procedure with a small risk of minor complications in elderly patients. Further randomized trials are required to confirm these results.
